# Relating proton LETd to biological response of parotid and submandibular glands using PSMA-PET in clinical patients

**DOI:** 10.1016/j.ctro.2024.100910

**Published:** 2025-01-09

**Authors:** Dirk Wagenaar, Vineet Mohan, Johannes A. Langendijk, Roel J.H.M. Steenbakkers, Wouter V. Vogel, Stefan Both

**Affiliations:** a Department of Radiation Oncology, University Medical Center Groningen, University of Groningen, Groningen, the Netherlands; b Department of Radiation Oncology, The Netherlands Cancer Institute, Amsterdam, the Netherlands

## Abstract

**Background and purpose:**

A recent study investigated the use of PSMA-PET in monitoring loss of secretory cells in salivary glands of head and neck cancer (HNC) patients. Previously, a dose–effect relation has been formulated to the PSMA-PET uptake in salivary glands. The aim of this study was to derive a proton RBE model from the PSMA-PET uptake in salivary glands after proton therapy of HNC patients.

**Materials and methods:**

Six patients treated with proton therapy were included. These patients received a PET-CT scan using ^68^Ga (N = 1) or ^18^F (N = 5) PSMA before treatment (baseline) and one month after the last fraction (follow-up). Physical dose (D), D·LET_d_ and the follow-up PSMA-PET scan were deformed to the baseline PET-CT using deformable image registration. Parotid and submandibular gland delineations were adjusted to include voxels which had an uptake of ≥ 5 g/ml in the baseline PSMA-PET scan.

**Results:**

The average RBE-LET slope was 0.075 [0.009; 0.125] (keV/μm)^-1^ (mean [95 %CI]) for parotid and submandibular glands combined. When analyzing parotid or submandibular glands separately the RBE-LET curve slope varies with two and five patients showing a positive RBE-LET slope when only analyzing parotid or submandibular glands respectively.

**Conclusion:**

Our study did not find clear evidence of an increased RBE in parotid and submandibular glands with increasing LET_d_. On average an LET_d_ effect was observed, however our sample size was too small to clearly define an RBE-LET relation. A larger cohort scanned at later time intervals could shed more light on this issue.

## Highlights

• PSMA-PET uptake decrease after treatment in parotid glands relates to radiation dose.
• The role of LET_d_ in PSMA-PET uptake decrease was studied.
• The role of LET_d_ in parotid glands might be smaller than predicted by preclinical experiments.
• A larger cohort scanned at later time intervals could be used to shed more light on this issue.

## Introduction

Proton therapy is a treatment modality with beneficial physical properties compared to conventional X-ray radiotherapy. The relative biological effectiveness (RBE) of protons is higher compared to X-rays and a uniform RBE factor of 1.1 is clinically advised [1]. However, the RBE is expected to rise as protons lose their energy resulting in a potentially higher biological effect at the end of proton range [2], [3]. Different variable RBE models have been proposed based on preclinical data with varying predictions [4], [5], but with a similar dependency on the dose-weighted average linear energy transfer (LET_d_) [6]. One potentially limiting factor is that these RBE models are based on preclinical data and that actual RBE is endpoint specific and likely to be different in patients than in cell lines [7]. For this reason, there is need for clinical validation of variable RBE models, as was underlined by both the radiobiology workgroup of the European Particle Therapy Network (EPTN) and a recent survey filled in by 25 European proton centers [8], [9].

Prostate-specific membrane antigen (PSMA)-positron emission tomography (PET) is typically used to detect prostate cancer cells [12], [13]. However, PSMA-PET has also been shown to visualize the density of acinar cells in salivary glands. The PSMA-PET uptake in salivary gland signal has been shown to decrease in areas receiving high radiotherapy dose, making it possible to quantify a dose–effect relation [13]. This potentially makes PSMA-PET a valuable instrument to quantify radiobiological damage on a voxel level. A multicenter clinical trial investigated this dose–effect relation in head and neck cancer (HNC) patients [13]. A subset of the included patients of this trial received proton therapy making it possible to investigate the RBE-LET relation.

Investigating the RBE-LET relation with a voxel-based analysis instead of a conventional normal-tissue complication probability (NTCP) model has several potential benefits. The number of datapoints per patient exceeds the order of thousands and the outcome metric is continuous instead of dichotomous. This is necessary, as a previous study showed conventional NTCP modelling is unlikely to provide evidence for the RBE-LET relation for HNC patients [14]. The aim of this study was to derive an RBE model from the relative decrease in PSMA-PET uptake in parotid and submandibular glands after proton therapy of HNC patients.

## Materials and methods

### Patients and treatment

We considered all proton therapy patients included in the “Dose-Effect Relation of Salivary Gland Irradiation” (clinical trials identifier: NCT03367780) study performed by the Netherlands Cancer Institute in collaboration with the University Medical Center Groningen [16]. The study protocol was reviewed by the Medical Ethical Committees of the Netherlands Cancer Institute and University Medical Center Groningen (registration number ABR NL 60569.031.17).

In the Netherlands, HNC patient selection for proton therapy is done according to the model-based selection method. In this method, both a conventional photon and a proton therapy treatment plan to the same prescription dose are made for every patient. HNC patients qualify for proton therapy if the expected benefit in terms of NTCP (ΔNTCP) exceeds 10 % for a single grade II complication, 15 % for the combined total of two grade II complications, 5 % for a single grade III complication, or 7.5 % for the combined total of two grade III complications [17].

In total, six of the included patients received proton therapy and were included in the current study. These patients were treated between April 2019 and January 2021. The relevant patient details and their received scans are listed in Table 1. One patient received a plan adaptation after the 8th fraction and other patients completed treatment on the original treatment plan.

**Table 1:** Patient specific scan timepoints and the.

Patient	Plan adaptation	Tracer	Injected dose [MBq]		
			Baseline	1-month	6-month
1	−	^86^Ga	50	50	50
2	Fx 8	^18^F	99	94	−
3	−	^18^F	93	91	−
4	−	^18^F	105	99	−
5	−	^18^F	107	110	−
6	−*	^18^F	101	91	−

−: Not applicable or missing.

*: Started palliative treatment 4-months after start of treatment.

Patients were treated with 70.00 Gy_RBE_ to the primary and 54.25 Gy_RBE_ to the prophylactic CTVs respectively in 35 fractions assuming a constant RBE of 1.1. Treatment plans were generated using robust optimization in the treatment planning system (TPS) RayStation 9A (RaySearch Laboratories, Stockholm, Sweden) using a 3-mm setup and 3 % range uncertainty setting [18], [19]. Robust evaluation was performed to assess adequate target coverage with the goal to cover 95 % of the target volumes with 98 % of their prescribed dose (i.e., V_95%_ > 98 % and D_98%_ > 95 %) in the voxel-wise minimum dose distribution [20]. Before delivery, each treatment plan was delivered in quality assurance mode, and the dose is recalculated based on the delivery log files on the planning CT using an independent Monte Carlo dose engine [21].

Patients’ head, neck and shoulders were immobilized with a 5-point mask (HP Pro, Orfit Industries, Wijnegem, Belgium). Daily cone-beam CT (CBCT) imaging was used to calculate a correction vector including shifts and rotations which was applied using a 6-D robotic couch. IMPT treatments were delivered at the UMCG proton therapy facility (Proteus ®Plus, IBA, Ottignies-Louvain-la-Neuve, Belgium). Patients received a weekly verification CT follow-up in treatment position to monitor the effect of anatomical changes on the dose distribution.

### PSMA PET-CT scans

Patients received a baseline scan before the start of treatment. Scans consisted of a PSMA PET-CT scan initially using 45–50 MBq of ^86^Ga and later 90–112 MBq ^18^F (Table 1). Two follow-up scans were planned one and six months after treatment completion. Incubation period was 45 min ± 5 min with 12 min of scan acquisition time split over two bed positions. The PET imaging was combined with a low dose CT scan.

### Imaging analysis

Clinical treatment plans were exported anonymized to a development build of RayStation based on v9R (8.99.30.71). The D and D·LET_d_ were recalculated in a 2 mm isotropic dose grid on the original planning CT using Monte Carlo with a 0.5 % statistical uncertainty per beam in RayStation. The LET_d_ calculations were validated in a previous study [22]. The D and D·LET_d_ distributions were deformably mapped to the PET-CT from before treatment (i.e., baseline). The delineations of the parotid and submandibular glands made on the planning CT were deformably registered to the baseline scan and adjusted to include the voxels within 6 mm of the original delineations with at least 5.0 g.ml standard uptake value (SUV) on the baseline PSMA scan. All available follow-up PET-CT scans were deformably registered to the baseline PET-CT scan. The D, D·LET_d_, baseline PSMA uptake and follow-up PSMA uptake were exported for every PET voxel within each parotid and submandibular gland for statistical analysis.

### Statistical analysis

Analyses were performed in Python SciPy v1.11.4 [23]. The follow-up PSMA uptake was fitted individually per patient using the dual annealing function to ensure a global minimum with a maximum of 10.000 iterations with the model:

$$\mathrm{PSM}A_{\mathrm{FU}}=PSMA_{0}\cdot \beta_{0}\cdot \exp\left({-\beta }_{1}\cdot Dose\cdot \left(1+\beta_{2}\cdot LET_{d}\right)\right)$$

Where PSMA_FU_ is the PSMA uptake at the one-month or six-month follow-up, PSMA_0_ is the PSMA uptake at baseline and β_0,_ β_1_ and β_2_ are the fit parameters. The β_0_ allows for universal scaling of the PSMA uptake which can account for dose independent factors at the time of the scan. The β_1_ describes the radiosensitivity and the β_2_ in describes the increase of RBE per unit LET_d_. The results of this fit can be used to formulate an RBE model of the form $\mathrm{RBE}=1.0+\beta_{2}\cdot LET_{d}$.

Analyses were performed on all parotid glands, on all submandibular glands and on both combined. Fitting the data for the types of glands separately allows for gland-specific fits of the radiosensitivity and RBE-LET slope. The slopes of the individual RBE-LET models were combined to estimate a population average slope with a 95 % confidence interval (95 %CI).

## Results

**Fig. 1:**
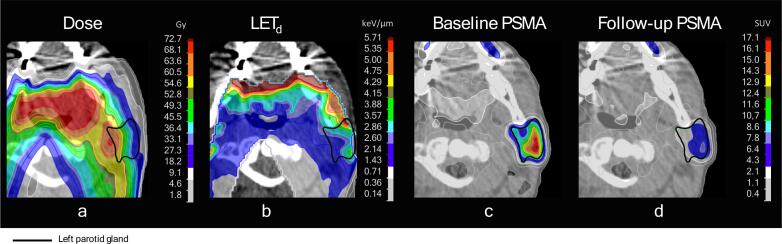
Visual example of used data and outcome. Shown are the results of the left parotid of patient 3. The physical dose distribution has no RBE factor applied. The follow-up PSMA scan was taken one month after proton therapy treatment. Some voxels in the shown parotid gland can be seen to receive equal amounts of physical dose (a), but different D⋅LET_d_ (b). If areas with higher D⋅LET_d_ but equal dose have a larger reduction in PSMA uptake (c and d), that would be indicative of an increased RBE for high LET protons. Dose (D) indicates physical dose (i.e., no RBE factor included).

The dose, LET_d_, baseline PSMA uptake and follow-up PSMA uptake of an example patient are shown in Fig. 1.

**Fig. 2:**
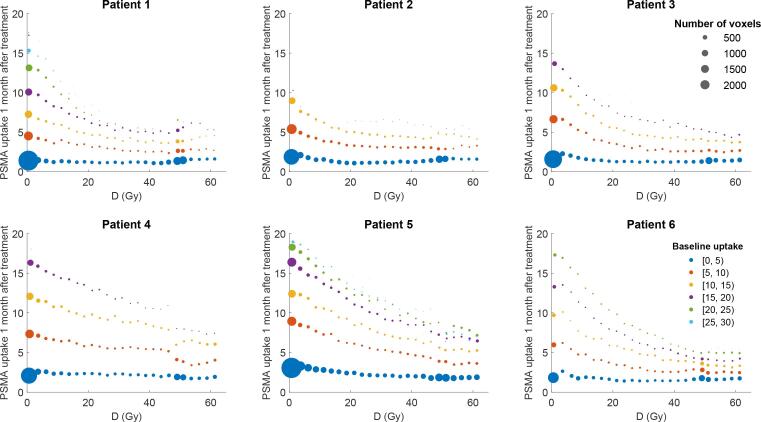
PSMA uptake one month after treatment for parotid and submandibular glands after proton therapy. Line plots of the number of voxels in the parotids and submandibular glands that receive a specific physical dose (in 2.5 Gy intervals) and the corresponding average PSMA-PET uptake 1 month after treatment in those voxels. The size of the points illustrates the number of voxels. The color of the points corresponds to the average PSMA-PET uptake from those voxels at baseline. Note that voxel sizes in the analysis are equal to the PET voxel sizes. Dose (D) indicates physical dose (i.e., no RBE factor included).

**Fig. 3:**
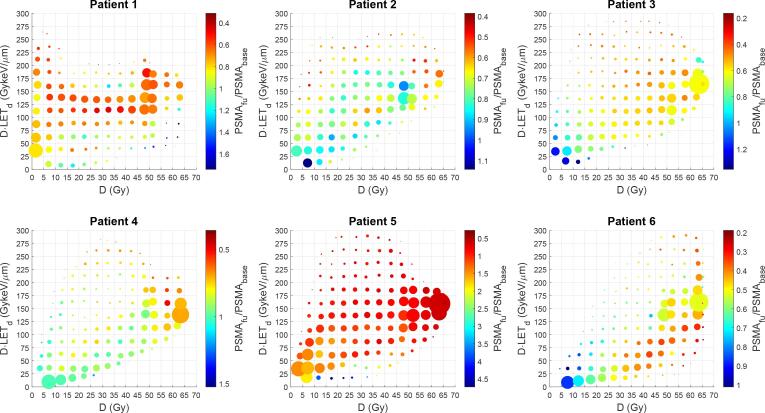
Relative PSMA uptake one month after treatment for the parotid and submandibular glands versus physical dose and LET. PET voxels were grouped into dose bins of 5.0 Gy and D⋅LET_d_ voxels of 25 GykeV/μm. The position of each data point indicates the average physical dose and D⋅LET_d_, the size indicates the number of voxels included and the color indicates the relative PSMA uptake (uptake one month after treatment divided by the baseline uptake). A low relative PSMA uptake (red) indicates more cellular damage, and a high value (blue) indicates less loss of secretory cells. If a physical dose–effect relation is present, data points on the right side of each graph should be more red. Similarly, if the RBE increased with dose, data points higher on the vertical axis should be more red. Datapoints in the lowest dose and D⋅LET_d_ were excluded as these are non-informative and contained between 19 %-38 % of the voxels. Dose (D) indicates physical dose (i.e., no RBE factor included). (For interpretation of the references to color in this figure legend, the reader is referred to the web version of this article.)

Scatter plots of the PSMA uptake one-month after treatment in the parotid glands versus physical dose are shown in Fig. 2 for all patients. Visual inspection shows an exponential decrease of PSMA uptake with increasing dose. Scatter plots of the relative PSMA uptake one-month after treatment versus physical dose and LET_d_ are shown in Fig. 3.

The results of the model fits are shown in Table 2. The model fits for parotid and submandibular glands show a positive RBE-LET slope value (β_2_) for five patients, but an inverted effect for one patient. The average RBE-LET slope was 0.075 [0.009; 0.125] (keV/μm)^-1^ (mean [95 %CI]). When analyzing the parotid and submandibular glands separately, the RBE-LET curve slope varies with two and five patients showing a positive RBE-LET slope when only analyzing parotid or submandibular glands, respectively.

**Table 2:** Fit results of PSMA uptake one-month after treatment in the parotid glands.

	Parotid and submandibular glands			Parotid glands			Submandibular glands		
Patient	β_0_	β_1_	β_2_	β_0_	β_1_	β_2_	β_0_	β_1_	β_2_
one-month follow up scan									
1	0.579	1.46e-4	0.000	0.584	2.22e-4	−0.017	0.491	5.43e-5	0.078
2	0.753	8.39e-5	0.206	0.778	1.36e-4	0.156	0.629	6.48e-5	0.059
3	0.869	1.37e-4	0.111	0.863	1.85e-4	−0.011	0.322	1.02e-5	−0.692
4	0.986	7.44e-5	0.143	0.979	8.30e-5	0.009	0.989	9.95e-5	0.099
5	0.928	1.42e-4	0.044	0.924	1.55e-4	−0.046	0.759	1.20e-4	0.089
6	0.791	2.37e-4	−0.057	0.799	2.68e-4	−0.109	0.763	1.48e-4	0.058
six-month follow up scan									
6	0.861	−3.29e-4	0.014	0.858	3.44e-4	0.007	0.468	2.32e-4	−0.062

For the fit function: $\mathrm{PSM}A_{\mathrm{FU}}=PSMA_{0}\cdot \beta_{0}\cdot \exp\left(-\beta_{1}\cdot Dose\cdot \left(1+\beta_{2}\cdot LET_{d}\right)\right)$.

**Fig. 4:**
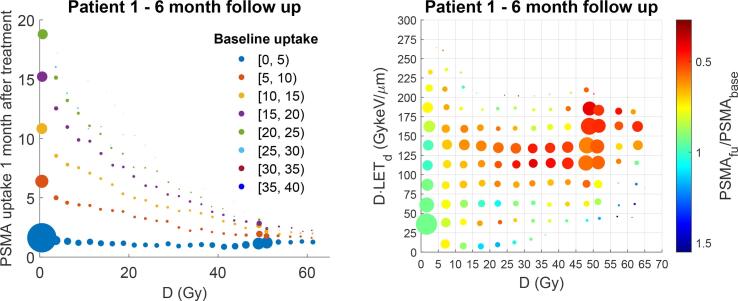
Results for the six-month follow up scan of patient 1. Analysis similar to Fig. 2 (left) and 3 (right) but for the six-month follow-up scan instead of the one-month follow-up scan. The fit parameters resulted in an RBE-LET curve slope of 0.014 (keV/μm)^-1^.

When repeating these analyses for the six-month follow up scan of one patient (Fig. 4), the RBE-LET curve slope was 0.014 (keV/μm)^-1^ when considering all salivary glands.

## Discussion

We observed no clear indications of an LET_d_ effect on relative PSMA uptake in parotid glands one month after treatment in all patients. Five out of six cases showed decrease in relative PSMA uptake with increasing LET_d_, but this effect was not clearly observed when analyzing parotid or submandibular glands separately. The population average RBE-LET slope was 0.075 [0.009; 0.125] (keV/μm)^-1^ (mean [95 %CI]).

Several explanations can be hypothesized for the lack of a clear effect. It is possible that our sample size was too small to accurately determine the RBE-LET curve slope for this endpoint. While each patient in principle has theoretically enough parotid gland voxels to determine an individual RBE-LET curve slope, interpatient differences may still require a larger patient cohort. The 95 %CI ranged from −0.009 to 0.125 (keV/μm)^-1^. Based on other studies, the expected slope is between 0.040 and 0.120 (keV/μm)^-1^ [3,11,29]. Our results seem to fall within this expected range. However, when analyzing the data for parotid and submandibular glands separately, the RBE-LET slope appears to vary.

If the slope for this endpoint is on the lower end of that range, it would still fall within our 95 %CI, in which case our low sample size could explain the lack of a clear effect. Additionally, the effect of LET_d_ might be more pronounced at later time points such as the six-month follow up scan. For the single patient in our dataset who had a six-month follow up scan the calculated RBE-LET curve slope was closer to the expected value based on the literature than the one-month follow up scan. A longer follow-up time might increase the effect of LET as the late response of tissue is associated with lower α/β values which are linked to a higher dependency of RBE on LET_d_ [5]. Therefore, our results might have been different if every patient had undergone a six-month follow up scan.

Earlier attempts have been successful in validating variable proton RBE in patients using voxel-based analyses in the brain [10], [11]. Bahn modeled the incidence of contrast-enhancing brain lesions in 110 proton therapy patients using physical dose, LET_d_ and distance to the ventricular system [10]. Their model was consistent with an RBE-LET curve slope of 0.10 (keV/μm)^-1^. This result was recently validated by a similar study by Eulitz et al. in 42 patients who found a similar slope of 0.12 (keV/μm)^-1^. These studies used a dichotomous primary endpoint: the presence or absence of a lesion on a contrast-enhanced T1-weighted MRI scan. An earlier study that uses a continuous endpoint like the present study was done by Skaarup et al.. They attempted to model the change in fractional anisotropy in six pediatric brain patients treated with proton therapy using physical dose and D·LET_d_, but their results were inconclusive [15]. Continuous endpoints can potentially provide more information regarding the damage in a voxel. However, the fact that both Skaarup et al. and the present study were unable to clearly show the RBE-LET relation in patients could indicate continuous voxel endpoints are currently not accurate enough or that the increase in RBE is limited.

Biological factors other than RBE might also play a role in PSMA uptake after radiation therapy [7]. Variable RBE models are largely based on clonogenic survival curves of tumor cells. The effect of LET_d_ can potentially be different for different cell types.

Additionally, our analysis treated parotid glands as homogeneous, but it is known that a sub region within the glands has a high density of stem cells [30]. Radiation dose to this stem cell region affects the salivary function of the entire gland [30,31]. Therefore, the modelling of voxel-wise response could potentially be improved by including such effects.

The dose and LET_d_ distributions considered in this work were calculated on the planning CT taken before the start of treatment. During treatment, errors in patient positioning and anatomical changes impact the dose and LET_d_ distributions. However, patient immobilization, daily verification and plan adaptation strategies were used to mitigate this effect. Even so, dose accumulation based on daily imaging could be used to more accurately estimate the actual given dose and LET_d_ [32,33].

Some limitations may have impacted our results. Our findings rely on accurate deformable image registration between PET-CTs. Misalignment of these scans could potentially result in large errors in the relative PSMA uptake. We only assumed a linear relation between RBE and LET. Additionally, our results could have been impacted by the low sample size and the relatively short follow-up with most patients receiving only one follow-up scan one month after treatment.

## Conclusions

Our study did not find clear evidence of an increased RBE in parotid and submandibular glands with increasing LET_d_. Our data suggests the role of LET_d_ in salivary glands might be very patient specific. On average an LET_d_ effect was observed, however our sample size was too small to clearly define an RBE-LET relation. A larger cohort scanned at later time intervals could shed more light on this issue.

## Ethics approval and consent to participate.

The Medical Ethics Committee of the Netherlands Cancer Institute (CCMO trial registration NL60569.031.17) approved the study protocol. The study was conducted in accordance with the Declaration of Helsinki. Written and oral informed consent was obtained from all patients prior to study entry.

## Availability of data and material.

The datasets generated and analyzed for this work may be available from the corresponding author on reasonable request.

## Funding

This work was supported by the Dutch Cancer Society KWF [Research Grant: 10606/2016–2].

## Declaration of Competing Interest

The authors declare that they have no known competing financial interests or personal relationships that could have appeared to influence the work reported in this paper.
